# Handheld Device for Selective Benzene Sensing over Toluene and Xylene

**DOI:** 10.1002/advs.202103853

**Published:** 2021-11-27

**Authors:** Ines C. Weber, Pascal Rüedi, Petr Šot, Andreas T. Güntner, Sotiris E. Pratsinis

**Affiliations:** ^1^ Particle Technology Laboratory Department of Mechanical and Process Engineering ETH Zurich Zurich CH‐8092 Switzerland; ^2^ Department of Chemistry and Applied Biosciences ETH Zurich Zurich CH‐8049 Switzerland; ^3^ Department of Endocrinology Diabetology and Clinical Nutrition University Hospital Zurich (USZ) and University of Zurich (UZH) Zurich CH‐8091 Switzerland

**Keywords:** aromatics, benzene sensing, electronics, environmental, gas sensors, nanotechnology

## Abstract

More than 1 million workers are exposed routinely to carcinogenic benzene, contained in various consumer products (e.g., gasoline, rubbers, and dyes) and released from combustion of organics (e.g., tobacco). Despite strict limits (e.g., 50 parts per billion (ppb) in the European Union), routine monitoring of benzene is rarely done since low‐cost sensors lack accuracy. This work presents a compact, battery‐driven device that detects benzene in gas mixtures with unprecedented selectivity (>200) over inorganics, ketones, aldehydes, alcohols, and even challenging toluene and xylene. This can be attributed to strong Lewis acid sites on a packed bed of catalytic WO_3_ nanoparticles that prescreen a chemoresistive Pd/SnO_2_ sensor. That way, benzene is detected down to 13 ppb with superior robustness to relative humidity (RH, 10–80%), fulfilling the strictest legal limits. As proof of concept, benzene is quantified in indoor air in good agreement (*R* ^2^ ≥ 0.94) with mass spectrometry. This device is readily applicable for personal exposure assessment and can assist the implementation of low‐emission zones for sustainable environments.

## Graphical abstract

A handheld detector is presented that quantifies benzene down to 13 ppb at 50% relative humidity and works even in real indoor air. This is enabled by superior benzene selectivity over most critical toluene and xylene provided by a flame‐made, catalytic WO_3_ filter prescreening a chemoresistive Pd/SnO_2_ sensor. As such, it is promising for routine benzene monitoring to identify emission hotspots.

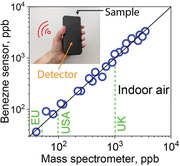

## 1 Introduction

Gaseous benzene is an environmental mutagen and highly toxic compound that induces hematological malignancies^[^ ^1^ ^]^ including multiple myeloma and leukemia.^[^ ^2^ ^]^ Frequently, accidents with benzene cause fatalities (e.g., in India in 2020^[^ ^3^ ^]^) and lead to severe drinking water contamination in the USA^[^ ^4^ ^]^ (2017) and China^[^ ^5^ ^]^ (2014). Therefore, it is a threat to environmental sustainability and occupational safety (estimated 1 012 500 workers were exposed to critical benzene levels in 2020^[^ ^6^ ^]^). This is recognized by strict exposure limits, for instance, an average of 100 ppb^[^ ^7^ ^]^ in the USA during 8 h workplace exposure, while the World Health Organization ^[^ ^8^ ^]^ even states that there is “no safe level” at all. Nevertheless, benzene is an inevitable chemical in the petroleum industry and contained in various consumer products (e.g., paint^[^ ^9^ ^]^ or glue^[^ ^10^ ^]^). Also, it occurs at elevated concentration frequently exceeding limits,^[^ ^11^ ^]^ for instance, at gas stations,^[^ ^12^ ^]^ in the vicinity of motorways^[^ ^13^ ^]^ and in cigarette smoke.^[^ ^14^ ^]^

Despite the requirement for benzene monitoring (e.g., norm 29 CFR 1910.1028 in the USA^[^ ^15^ ^]^) in affected industries (e.g., coke and coal chemicals, petroleum refineries, petrochemicals), today, it is measured mostly occasionally by onsite sampling and offsite quantification in external laboratories (e.g., mass spectrometry‐based).^[^ ^16^ ^]^ Onsite analysis is possible with portable gas chromatographs coupled to photo ionization detectors (e.g., Draeger X‐pid 8500^[^ ^17^ ^]^) or colorimetric tubes (e.g., by Draeger^[^ ^18^ ^]^). However, these can cost several thousand US‐Dollars , require ≈10 min for start‐up^[^ ^17^ ^]^ or are single‐use only.^[^ ^18^ ^]^ Most importantly, these procedures hardly allow personalized benzene exposure assessment to track peak concentrations and warn users at the point‐of‐care. This might be realized with low‐cost chemical (e.g., optical or chemoresistive) sensors integrated into handheld^[^ ^19^ ^]^ devices. To date, various sensors are promising (**Table** **1**) that can detect benzene down to 2.5 ppb^[^ ^20^ ^]^ within few minutes. Yet, such sensors are compromised by insufficient benzene selectivity, a recurring issue with air quality sensors,^[^ ^21^ ^]^ in particular over chemically similar xylene and toluene (i.e., aromatics) that can be present at an order of magnitude higher concentration than benzene.^[^ ^22^ ^]^

**Table 1:** Selectivity and lowest quantified concentration of low‐cost benzene detectors

Material				RH [%]	Selectivity (*S*_benzene_/*S*_analyte_,–)											LOQ^a^ [ppm]	Ref.
					Toluene	Xylene	CO	Ethylbenzene	Acetone	Ethanol	Methanol	Isoprene	Hydrogen	Acetaldehyde	NO_2_		
Optical			Planar Bragg grating	–	0.4	0.1	–	–	–	–	–	–	–	–	–	1000	^[^ ^73^ ^]^
Chemoresistive	Metal oxide		Pd/SnO_2_–ZnO	–	11.5	–	7.7	–	–	–	–	–	–	–	–	0.1	^[^ ^38^ ^]^
			SnO_2_–Cu_2_O	–	1.1	–	–	–	–	–	–	–	–	–	8	10	^[^ ^35^ ^]^
			SnO_2_/Co_3_O_4_	–	–	–	5.8	–	3.6	–	–	–	–	–	–	0.1	^[^ ^74^ ^]^
			Pd‐TiO_2_/MoS_2_	–	–	–	7	–	–	–	–	–	–	–	–	0.1	^[^ ^75^ ^]^
			Pd/SnO_2_	50^b^/80^c^	0.9	0.9	38	2.3	1.5	2	4.8	1.4	9.3	3.2	10	0.013	^i^
	Carbon composites		CoPP^d^‐TiO_2_	–	0.5	0.5	5.9	3.6	3.6	–	–	–	–	–	0.8	0.005	^[^ ^43^ ^]^
			Pd‐rGO^e^/ZnO	–	1	–	1.4	–	–	–	–	–	–	–	–	1	^[^ ^37^ ^]^
			Au/MWCNT^f^	0^b^/60^c^	7	30	>100	–	–	1000	–	–	–	–	–	0.0025	^[^ ^20^ ^]^
			Pentiptycene/SWCNT^f^	0^b^/70^c^	0.2	0.5	–	–	6.9	–	15	–	–	–	–	5	^[^ ^36^ ^]^
	Sorption filter		SnO–SnO_2_ + GC^g^ column	–	–	–	∞	–	–	–	–	–	–	–	–	0.005	^[^ ^44^ ^]^
	Catalytic filter	Overlayer	Co_3_O_4_ filter + Pd/SnO_2_ sensor	–	2.6	5.3	2.7	–	–	5.2	–	–	–	–	–	0.25	^[^ ^25^ ^]^
			Rh‐TiO_2_ filter + SnO_2_ sensor^h^	0^b^/80^c^	3.9	8.4	21	–		21	–	–	–	–	–	1	^[^ ^26^ ^]^
			Pt/Al_2_O_3_ filter + WO_3_ sensor	15^b,c^	–	–	–	–	–	5.8	–	–	–	–	0.2	1	^[^ ^24^ ^]^
		Bed	WO_3_ filter + Pd/SnO_2_ sensor	50^b^/80^c^	>200										30	0.013	^i^

^a^LOQ: limit of quantification; ^b^RH level applied for LOQ and selectivity measurement; ^c^Highest RH tested; ^d^CoPP: cobalt porphyrin; ^e^rGO: reduced graphene oxide; ^f^S‐/MWCNT: single/multiwall carbon nanotubes; ^g^GC: gas chromatography; ^h^2Rh‐TiO_2_/SnO_2_ at 325 °C, ^i^This work.

A promising strategy to improve benzene selectivity are catalytic filters that are introduced upstream of sensors to convert interferants to nonresponsive species by chemical reaction.^[^ ^23^ ^]^ Such filters were explored for benzene detection as catalytic overlayers on top of sensing films (i.e., Pt/Al_2_O_3_^[^ ^24^ ^]^, Co_3_O_4_^[^ ^25^ ^]^ and Rh/TiO_2_^[^ ^26^ ^]^). This improved benzene selectivity toward ethanol^[^ ^24^, ^25^, ^26^ ^]^ and CO,^[^ ^25^, ^26^ ^]^ but was limited for toluene (Table 1). Various parameters influence catalyst selectivity (e.g., crystal/particle size, shape, exposed facets^[^ ^27^ ^]^ and acid–base and redox properties^[^ ^28^ ^]^), where surface acidity is often a critical one.^[^ ^29^ ^]^ Specifically for aromatics, their interaction with acidic sites is strongly affected by the number of methyl groups (e.g., 0 for benzene, 1–2 for toluene and xylene) that donate electrons to the aromatic ring.^[^ ^30^ ^]^

Here, we report a fully integrated and portable detector for selective benzene monitoring in indoor air. It combines a catalytic packed bed filter of WO_3_ nanoparticles featuring high Lewis acidity,^[^ ^31^ ^]^ with a highly sensitive but nonspecific^[^ ^32^ ^]^ Pd/SnO_2_ sensor. The detector is evaluated first on various indoor air‐relevant analytes (i.e., *meta*‐xylene (*m*‐xylene), toluene, acetone, acetaldehyde, isoprene, methanol, ethanol, CO, hydrogen, and ethylbenzene) and their gas mixtures as well as validated by proton‐transfer‐reaction time‐of‐flight mass spectrometry (PTR‐ToF‐MS). Next, the robustness to RH is assessed. To better understand the catalyst performance, its bulk and surface properties are investigated with X‐ray diffraction, transmission electron microscopy (TEM) and Fourier transform infrared spectroscopy (FTIR) after pyridine adsorption. As a proof‐of‐concept, the device is tested with benzene‐spiked indoor air.

## 2 Results and Discussion

### 2.1 Selective and Rapid Benzene Sensing Down to ppb with Humidity Robustness

**Figure 1:**
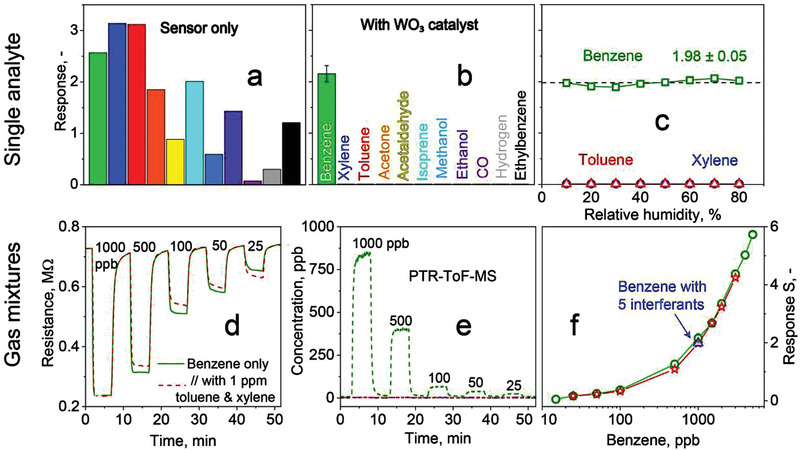
Selective benzene sensing enabled by catalytic WO_3_ filter. Chemoresistive response of Pd/SnO_2_ to 1 ppm benzene, *m*‐xylene, toluene, acetone, acetaldehyde, isoprene, methanol, ethanol, CO, hydrogen, and ethylbenzene when prescreened by a) inactive (i.e., at room temperature; Figure S3a, Supporting Information) and b) active (240 °C) catalytic WO_3_ packed bed at 50% RH. c) Responses to 1 ppm toluene, *m*‐xylene and benzene at 10–80% RH. d) Sensor resistance upon exposure to 25–1000 ppb benzene as single analyte (solid line) and in mixtures with 1 ppm (each) toluene and *m*‐xylene (dashed line). e) Corresponding mixture benzene (green), toluene (red) and *m*‐xylene (blue) concentrations at the packed bed's outlet measured by PTR‐ToF‐MS. Note that the lines for toluene and *m*‐xylene are overlapping. f) Sensor responses to 15–5000 ppb of benzene alone (circles) and in mixtures with 1 ppm (each) toluene and *m*‐xylene (stars), as well as with five interferants (toluene, *m*‐xylene, acetaldehyde, CO, acetone, each 1 ppm, triangle). Error bars at 1 ppm benzene in (b) and (f, hidden behind symbol) correspond to *n* = 3 identically produced WO_3_ filters and Pd/SnO_2_ sensors.

**Figure** **1**a shows the response of the Pd/SnO_2_ sensor at 50% RH when exposed to 1 parts per million (ppm) benzene and several key interferants from various chemical families (e.g., aromatics, ketones, aldehydes, alcohols, and inorganics) typically contained in indoor and outdoor environments. The sensor exhibits similar responses to most analytes and particularly to benzene (2.8), *m*‐xylene (3.1), and toluene (3.1). Such nonselective behavior is common for SnO_2_‐based detectors,^[^ ^33^ ^]^ thus this sensor alone is not capable to distinguish benzene, especially from *m*‐xylene and toluene.

When, however, a packed bed of WO_3_ particles (at 240 °C) prescreens that sensor, it turns it selective to benzene (Figure 1b). In fact, then only benzene is detected (response: 2.1), while all interferant responses are mitigated effectively (< 0.02). This results in unprecedented benzene selectivity to all analytes (>200), including chemically similar aromatics like toluene and *m*‐xylene. This is valid also for CO even at concentrations up to 25 ppm (Figure S1 in the Supporting Information, as might be present in ventilated underground garages^[^ ^34^ ^]^) and for oxidizing gases like NO_2_ even in mixtures with benzene with sensor responses ≤±0.1 (Figure S2, Supporting Information). Note that the WO_3_ filter reduced also the benzene response by 23% suggesting its partial conversion (Figure 1a vs Figure 1b). Nevertheless, this does not affect the sensor's ability to detect even low ppb of benzene, as will be elaborated below. The performance of the present detector is reproducible, as demonstrated with three identically produced sensors and filters that showed a response variation of <10% (error bars in Figure 1b).

The excellent selectivity of the present detector outperforms other low‐cost ones that had been designed specifically for benzene (Table 1). For instance, most metal oxide‐ or carbon‐based composite detectors (e.g., SnO_2_–Cu_2_O,^[^ ^35^ ^]^ Pentiptycene/SWCNT,^[^ ^36^ ^]^ or Pd‐rGO/ZnO^[^ ^37^ ^]^) were interfered by toluene and xylene featuring higher or similar responses to benzene. Even filter‐enhanced sensors (e.g., similar Pd/SnO_2_ with a Co_3_O_4_ overlayer^[^ ^25^ ^]^) showed only moderate (≤8.4) selectivities to these interferants. Also CO interfered most of these sensors (e.g., Pd/SnO_2_–ZnO^[^ ^38^ ^]^) where only SnO_2_ with an Rh‐TiO_2_ overlayer^[^ ^26^ ^]^ and Au/MWCNT^[^ ^20^ ^]^ detectors showed promising benzene selectivity (21 and >100, respectively), however, this has to be confirmed in the presence of realistic humidity.

Next, the detector was assessed for its humidity robustness to typical fluctuations in indoor and outdoor air. The benzene response hardly changed (i.e., 2 ± 0.1, squares in Figure 1c) between 10% and 80% RH. Moreover, the benzene selectivity over toluene (triangles) and *m*‐xylene (circles) is preserved consistently, as both compounds are not picked up by the detector over the entire RH range, similar to heated^[^ ^39^ ^]^ and room temperature^[^ ^40^ ^]^ catalytic filters preceding sensors for selective acetone detection in human breath.^[^ ^41^ ^]^ This is a distinct advantage for environmental benzene monitoring over other sensors susceptible to humidity changes: For instance, Au/MWCNT suffered from a large loss in benzene response (i.e., 80%) when increasing the RH from 10 to 60%.^[^ ^20^ ^]^ This was less pronounced for Rh/TiO_2_ catalyst‐screened SnO_2_ sensors, where increasing the RH from 41% to 80% led only to a 30% response loss.^[^ ^26^ ^]^ Only Pentiptycene/SWCNT sensors showed reasonable RH robustness between 3% and 70% RH (<10% deviation), measured, however, at rather high (100 ppm) benzene concentrations.^[^ ^36^ ^]^

Since legal exposure limits for benzene can be as low as 50 ppb (i.e., European Union^[^ ^42^ ^]^), the present detector was evaluated with exposure to 25–1000 ppb benzene at 50% RH (Figure 1d, solid line). Most importantly, the detector measures even the lowest concentrations with high signal to noise ratio (i.e., 12 at 25 ppb) and clearly distinguishes these concentrations, thus fulfilling even the strictest guidelines. Lower concentrations were detected only with CoPP‐TiO_2_,^[^ ^43^ ^]^ Au/MWCNT,^[^ ^20^ ^]^ and a SnO–SnO_2_ sensor coupled to a GC column,^[^ ^44^ ^]^ though in dry air (Table 1). In addition, the present detector features fully reversible behavior and fast response time and recovery time (i.e., 36 and 47 s at 100 ppb, respectively; Figure 1d). This enables on‐site benzene monitoring at high frequency to recognize harmful concentrations and warn the operator immediately, a major advantage over established off‐site analyses.^[^ ^16^ ^]^

### 2.2 Performance in Gas Mixtures

To challenge the detector further, its performance was assessed in gas mixtures. Most importantly, when tested on 25–1000 ppb of benzene in the presence of 1000 ppb (each) toluene and *m*‐xylene (dashed line, Figure 1d), an almost identical (average difference <2.5%) resistance profile to the benzene alone (solid line) is obtained. This confirms the detector's excellent benzene selectivity in mixtures even with orders of magnitude higher and most critical interferant concentrations. We validated these results by simultaneous high‐resolution PTR‐ToF‐MS measurement that indicated complete removal of toluene and *m*‐xylene at the outlet of the WO_3_ filter (Figure 1e) while the benzene was converted only partially (loss 22.1 ± 6.6%), in agreement with Figure 1b. Finally, we tested the detector at 1000 ppb benzene in the presence of five critical interferants (toluene, *m*‐xylene, acetaldehyde, CO, acetone; each at 1000 ppb). Most remarkably, the sensor response (triangle, Figure 1f) is quite similar (≤7% deviation) to the aforementioned measurements with benzene only (squares) and in the presence of toluene and *m*‐xylene (stars), showing even better the detector's outstanding selectivity.

### 2.3 Catalyst Characterization and Mechanism

**Figure 2:**
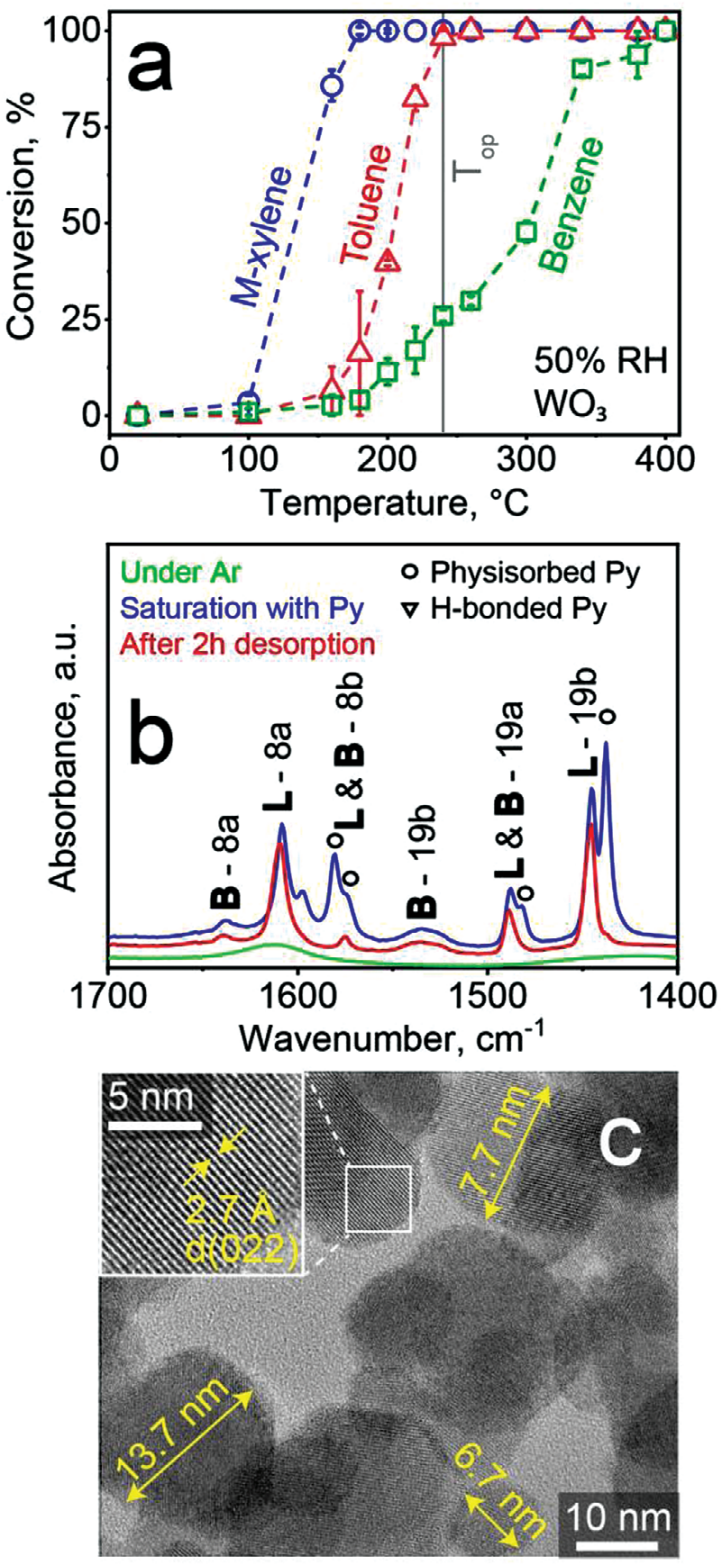
Catalytic and material characterization of WO_3_ nanoparticles. a) Catalytic conversion of 1 ppm *m*‐xylene (circles), toluene (triangles) and benzene (squares) over WO_3_ at 50% RH, as measured by PTR‐ToF‐MS. Error bars correspond to *n* = 3 identically prepared WO_3_ particle filters (some bars are smaller than their symbols). b) Infrared spectrum of such WO_3_ nanoparticles under Argon, after pyridine saturation and 2 h desorption. Nanoparticles were pretreated at 150 °C for 3 h in vacuum. Characteristic vibrations modes of Brønsted (B) and Lewis (L) acid sites are indicated together with physisorbed (circles) and H‐bonded pyridine (triangles). c) HRTEM bright field image of the flame‐made WO_3_ particles. Diameters of some particles are indicated. Inset shows magnification of selected area (box) with lattice fringes labeled by respective Miller indices.

The role of the WO_3_ filter is best understood by investigating first its catalytic reactivity toward *m*‐xylene, toluene and benzene with high resolution PTR‐ToF‐MS (**Figure** **2**a). *M*‐xylene starts reacting already at 100 °C and is removed completely at 180 °C. For toluene, conversion takes place at higher temperatures (i.e., 160–240 °C) while for benzene, it occurs even higher (i.e., between 180 and 400 °C). As a result, at 240 °C (gray line, T_op_), this catalyst removes *m*‐xylene and toluene quite selectively over benzene, that remained mostly unscathed (i.e., 26% ± 1.6% benzene loss), in agreement with the sensor results in Figure 1b. We confirmed this also for acetaldehyde, acetone and ethanol with the PTR‐ToF‐MS and the sensor (Figure S3, Supporting Information) that were removed completely at 240 °C. It is worth noting that the catalyst preserves its selectivity also when reducing the filter loading (i.e., from 81 to 40 mg; Figure S4, Supporting Information) and maintains its stability upon storage in room air for, at least, 100 days (Figure S5, Supporting Information), making it promising for compact integration (as demonstrated below) and prolonged use, respectively.

Acid–base properties of metal oxides are crucial when studying catalyst adsorption and selectivity behavior in catalytic oxidations.^[^ ^28^ ^]^ Therefore, the surface chemistry of the WO_3_ nanoparticles is characterized by FTIR with pyridine as probing molecule (Figure 2b). Two dominant bands at 1445 cm^−1^ (characteristic vibration mode *ν*_19b_) and 1609 cm^−1^ (*ν*_8a_) indicate strong Lewis bound pyridine.^[^ ^31^ ^]^ In contrast, bands attributed to Brønsted sites^[^ ^31^ ^]^ at 1641 cm^−1^ (*ν*_8a_) and 1533 cm^−1^ (*ν*_19b_) are very weak, indicating a low concentration of such sites, corresponding well with reported ammonia temperature programmed desorption (TPD) measurements.^[^ ^45^ ^]^ Note that the bands at 1489 cm^−1^ (*ν*_19b_) and 1575 cm^−1^ (*ν*_8a_) may be associated to both Brønsted and Lewis acid sites^[^ ^46^ ^]^ or H‐bonded pyridine.^[^ ^47^ ^]^ Also particle pretreatment at 400 °C for 3 h in vacuum hardly affected Lewis and Brønsted acidic sites (Figure S6 in the Supporting Information, showing also the complete adsorption and desorption profiles). Hence, Lewis acid sites are abundantly present on the surface of WO_3_ and quite likely play a major role in adsorption and activation of the reactants.^[^ ^30^ ^]^

In fact, *π*‐electron interactions of aromatic rings with Lewis acid sites^[^ ^48^ ^]^ are stronger for toluene^[^ ^29^ ^]^ and *m*‐xylene^[^ ^49^ ^]^ than benzene due to their methyl groups donating electrons to the ring,^[^ ^50^ ^]^ that correlates with the onset of their conversion (Figure 2a). Therefore, the excellent benzene selectivity over toluene and *m*‐xylene (Figure 1) of the detector may be associated to the high Lewis acidity of the WO_3_ nanoparticles. Note, however, that other factors (e.g., morphology,^[^ ^53^ ^]^ crystal phase and exposed facets,^[^ ^51^ ^]^ defects,^[^ ^52^ ^]^ humidity,^[^ ^53^ ^]^ etc.) can affect WO_3_ catalyst selectivity as well. In fact, altering crystal size, composition and SSA of the WO_3_ catalyst by annealing at 300 and 700 °C for 1 h (XRD patterns, SSA, and TEM images; Figures S7 and S8, Supporting Information) deteriorated the benzene selectivity (Figure S9, Supporting Information).

Moreover, the WO_3_ catalyst features excellent selectivity to critical confounders of different chemical families such as acetone, which is primarily converted on Lewis acid sites as well.^[^ ^54^ ^]^ For comparison, we investigated the catalytic properties of rather basic^[^ ^55^ ^]^ ZnO nanoparticles (specific surface area (SSA) and XRD pattern in Figure S7 (Supporting Information) and high‐resolution transmission electron microscopy (HRTEM) image in Figure S10c in the Supporting Information). The FTIR characterization with pyridine adsorption reveals Lewis and no Brønsted acid sites (Figure S10a,b, Supporting Information), in agreement with literature on pyridine^[^ ^56^ ^]^ and temperature programmed desorption (TPD) of ammonia^[^ ^45^ ^]^ measurements. Now, acetone is converted at significantly higher temperatures (as had been exploited previously for its selective sensing^[^ ^57^ ^]^) that deteriorates benzene selectivity (Figure S10d, Supporting Information).

Finally, we investigated the bulk properties of the WO_3_ nanoparticles. These feature rather spherical shape with a geometric mean of 14.9 nm and standard deviation of 1.4 (Figure S11, Supporting Information), as shown with HRTEM (Figure 2c). The clearly visible lattice fringes (inset) indicate high crystallinity and the 2.7 Å spacing fits well to the (022) plane of *γ*‐ or *ε*‐WO_3_. This is in agreement with X‐ray diffraction (XRD; Figure S7, Supporting Information, blue line), revealing the presence of both *γ*‐ and *ε*‐WO_3_ with crystal sizes of 19.9 and 17.4 nm, respectively, in line with literature.^[^ ^58^ ^]^ Such small particles with large surface area (i.e., 44 m^2^ g^−1^) are desired for high catalyst reactivity.

### 2.4 Handheld Device Quantifies Benzene in Spiked Indoor Air

**Figure 3:**
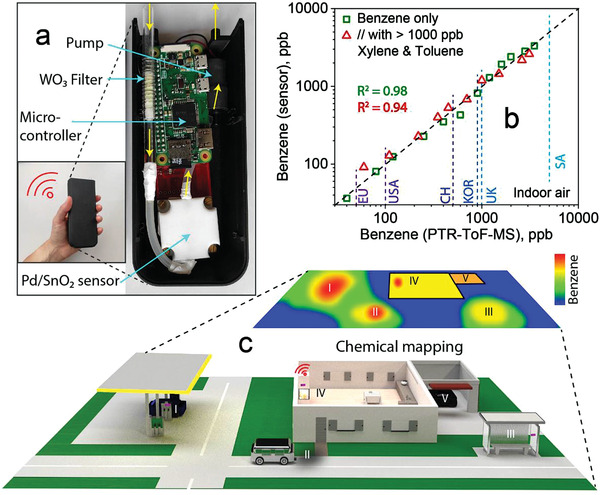
Indoor air measurements with handheld device. a) Fully integrated detector (inset) comprising the catalytic filter, a Pd/SnO_2_ sensor enclosed in a white Teflon chamber, a pump and a microcontroller. b) Scatter plot of benzene concentrations measured in indoor air spiked with 40–3500 ppb benzene alone (squares) and in the presence of toluene and *m*‐xylene (each >1000 ppb, triangles), as measured with the handheld detector and bench‐top PTR‐ToF‐MS. Various national exposure guidelines (i.e., EU,^[^ ^42^ ^]^ USA,^[^ ^7^ ^]^ CH,^[^ ^76^ ^]^ KOR,^[^ ^77^ ^]^ UK,^[^ ^78^ ^]^ and SA^[^ ^79^ ^]^) are indicated. c) Envisioned application of the mobile detector to track benzene concentrations at fuel stations, buildings and roads. Interconnecting such devices and wireless communication (i.e., Internet‐of‐things networks) to data clouds enables chemical mapping to identify emission “hotspots.”

The detector's immediate practical impact is demonstrated with a fully integrated, compact (i.e., 13.5 × 4 × 5.5 cm) and light‐weight (132 g) device hosting miniaturized versions (for details, see the Experimental Section) of the catalytic WO_3_ filter and the Pd/SnO_2_ microsensor together with a small pump to facilitate indoor air sampling (**Figure** **3**a). A microcontroller on a printed circuit board (PCB) operates the device, reads out the sensor resistance and can communicate the data wirelessly to a computer or smartphone for analysis and visualization.^[^ ^19^ ^]^ This device contains mostly commercially available components, thus can be produced at low‐cost in large quantities.

As proof of concept, the device was tested with 40–3500 ppb benzene‐spiked indoor air spanning the entire range of legal limits of six countries/regions (indicated by vertical dashed lines in Figure 3b). Note that spiking is a standard practice^[^ ^59^ ^]^ to simulate the complex conditions of real indoor air with its usually >250 compounds.^[^ ^60^ ^]^ Remarkably, the handheld device quantifies benzene accurately over more than two orders of magnitude, in good agreement (*R* ^2^ = 0.98) with PTR‐ToF‐MS as indicated by the dashed line (Figure 3b). This was hardly changed even when introducing additional toluene and *m*‐xylene (1 ppm each, triangles, *R* ^2^ = 0.94), or by significant variations of RH (e.g., 24.5%–37.6%; Figure S12a, Supporting Information), ethanol (48–920 ppb; Figure S12b, Supporting Information) and acetone (40–150 ppb) during these measurements. Most importantly, the bias of the detector is only 2% and the precision is 16% over the entire range (Figure S13, Supporting Information), enabling accurate benzene quantification, for instance, between 85 and 118 ppb at the USA limit (100 ppb). This is sufficient to identify hazardous indoor air conditions.

## 3 Conclusions

A low‐cost, user‐friendly and compact (fits in palm of hand) detector for accurate benzene quantification in indoor air was presented. To date, weak benzene selectivity had been a major bottleneck for such sensors that was overcome here by the rational design of a catalytic filter of WO_3_ nanoparticles that removed interferants selectively over benzene, probably due to high Lewis acidity. This is even valid for chemically similar toluene and *m*‐xylene, as confirmed in gas mixtures/real indoor air and validated (*R* ^2^ ≥ 0.94) by PTR‐ToF‐MS. The present device is capable of detecting benzene down to ppb concentrations with high robustness to humidity, meeting even the strictest national exposure guidelines.

As a result, this detector fulfils an urgent need for validated air quality trackers^[^ ^21^ ^]^ with high potential for on‐site indoor (e.g., from smoke, paint, or glue) and outdoor (e.g., at gas stations or high traffic roads) measurements to indicate harmful concentrations. We envision the device's application also as exposure patch for personalized air quality monitors to enhance occupational safety in critical industries by warning users/operators at the point of care. Given today's efforts toward interconnected (IoT) networks,^[^ ^61^ ^]^ such detectors could enable the chemical mapping of benzene to localize^[^ ^62^ ^]^ emission hotspots (Figure 3c) and assist policymakers in creating sustainable environments.

## 4 Experimental Section

### Catalyst Fabrication and Characterization

Catalytic WO_3_^[^ ^63^ ^]^ and ZnO^[^ ^64^ ^]^ nanoparticles were prepared by flame spray pyrolysis (FSP) of precursor solutions that were injected at a rate of 5 mL min^−1^ through a nozzle and dispersed with 5 L min^−1^ oxygen while maintaining a pressure drop of 1.7 bar. A ring‐shaped flow of premixed methane and oxygen (i.e., 1.2 and 3.2 L min^−1^, respectively) ignited and sustained the flame. Nanoparticles were collected 50 cm above the nozzle on a cooled glass‐fiber filter (GF‐6 Hahnemuehle, 257 mm diameter) through a vacuum pump (Seco SV 1025 C, Busch, Switzerland). The particles were removed with a spatula, sieved (250 *µ*m mesh) and annealed for 1 h at 500 °C, unless specified differently, in an oven (Carbolite Gero GmbH, 30–3000 °C).

Particle morphology was investigated by HRTEM imaging. Therefore, nanoparticles were dispersed in ethanol and deposited onto a perforated carbon foil supported on a copper grid. A double‐corrected microscope JEM‐ARM300F (GrandArm, JEOL) was used to obtain the high‐resolution images at 300 kV. The crystallinity and phase compositions were assessed by XRD with a Bruker AXS D8 Advance diffractometer operated at 40 kV and 30 mA. The recording was done at 2*θ* between 15° and 70° with a step size of 0.011° and a speed of 0.0057° s^−1^. Crystal phases were identified with the software Bruker Diffrac.eva V3.1 with reference parameters of monoclinic (PDF 83‐0950), orthorhombic WO_3_ (PDF 20‐1324) and wurtzite ZnO (PDF 41‐1426). The software TOPAS 4.2 was used to calculate crystal sizes with the Rietveld refinement method. The specific surface area (SSA) was determined with the Brunauer–Emmett–Teller (BET) method. Thereby, nanoparticle powders were degassed with N_2_ for 1 h at 150 °C before N_2_ adsorption on a Micromeritics Tristar II Plus.

The Lewis and Brønsted acid sites on WO_3_ and ZnO nanoparticles were investigated by FTIR spectroscopy with pyridine serving as probing molecule.^[^ ^65^ ^]^ First, nanoparticles were transferred into a static calcination reactor, heated to 150 or 400 °C in air for 3 h and evacuated under high vacuum (10^−5^ mbar, 12 h). Subsequently, surface defects were quenched by introducing O_2_ at 60 mbar (Pan Gas, purified over activated 3 Å molecular sieves), as such defects are unlikely to be present during catalytic experiments in synthetic air. Thereafter, 8.8 mg of WO_3_ or 7.7 mg of ZnO were pressed into a pellet (under high vacuum and at room temperature) and placed inside an IR cell with CaF_2_ windows. IR spectra (Nicolet 6700 FT‐IR, Thermo Scientific) were recorded after exposing the pellet to argon (purified, <1 ppm H_2_O, <1 ppm O_2_). Then, the argon atmosphere was removed, a small amount of pyridine was introduced, and the IR spectrum was recorded. The amount of pyridine was increased until saturation was reached, followed by pyridine desorption under isothermal conditions. Arising bands that occur upon exposure of argon and pyridine were correlated to vibration modes and analyzed in detail for the spectrum (1700–1400 cm^−1^) where Lewis and Brønsted acid site peaks appear. All measurements were carried out under isothermal conditions with a pellet/cell saturated by pyridine. Also, detector‐saturation effects are unlikely given the low value of absorption in the IR spectra (Figures S6 and S10, Supporting Information). These precautions should allow a semi‐quantitative analysis of the present sites by Lambert–Beer law. Note that pyridine (VWR International, ≥99%) was dried with CaH_2_ (Sigma‐Aldrich, ≥97%) and degassed on a high‐vacuum line with three freeze‐pump thaw cycles prior to the experiments.

The catalytic setup comprised a gas delivery system^[^ ^66^ ^]^ that was connected to the catalyst and bench‐top PTR‐ToF‐MS 1000 (Ionicon) through inert and heated Teflon tubing.^[^ ^57^ ^]^ The gas delivery system comprised high‐precision mass flow controllers (Bronkhorst) that dosed the analytes *m*‐xylene (10 ppm), toluene (10 ppm), benzene (9 ppm), acetone (18 ppm), acetaldehyde (17 ppm) and ethanol (14 ppm) into a dry synthetic air stream (all Pan Gas in synthetic air, C*_n_* H*_m_* and NO*_x_* ≤100 ppb). Humidity was admixed by bubbling dry synthetic air through ultrapure water (Milli‐Q A10, Merck, Switzerland) inside a 125 mL glass bottle (Drechsel bottle, sintered glass frit, Sigma‐Aldrich) to reach a total flow rate of 150 mL min^−1^, as monitored with a humidity sensor (SHT2x, Sensirion AG). The catalyst consisted of 81 mg WO_3_ or 180 mg ZnO (equivalent to 3.6 m^2^ total surface area) fixated inside a quartz glass reactor (inner diameter 4 mm) using quartz glass and quartz sand. The packed beds had a length of ≈2 cm and were checked visually for voids prior to measurements.

When placed inside an oven (Nabertherm, P320), the catalytic conversion was measured at the exhaust with a PTR‐ToF‐MS using H_3_O^+^ ions with a drift voltage, temperature and pressure of 600 V, 60 °C and 2.3 mbar, respectively. Analyte concentrations were determined at mass‐to‐charge (*m*/*z*) ratios of 45.03 (acetaldehyde), 47.05 (ethanol), 59.05 (acetone), 79.05 (benzene) 93.06 (toluene), and 107.16 (*m*‐xylene). All analytes were three‐point‐calibrated over the relevant range with the above standards prior to the measurements. Analyte conversion was calculated from the analyte (*i*) concentration at the catalyst outlet (*c*_out_) and inlet (*c*_in_)

(1) $$\mathrm{Conversion}=\left(1-\frac{c_{\mathrm{out},i}}{c_{\mathrm{in},i}}\right)$$

### Sensing Measurements

The sensor was based on 0.5 mol% Pd‐doped SnO_2_ nanoparticles prepared by the above FSP reactor through direct deposition^[^ ^67^ ^]^ onto alumina (15 × 13 × 0.8 mm^3^) substrates with interdigitated (spacing 350 µm) Pt electrodes, following a precursor formulation specified elsewhere.^[^ ^32^ ^]^ After particle deposition, the sensor was annealed for 5 h at 500 °C. This sensor was mounted on a Macor holder inside a Teflon chamber^[^ ^68^ ^]^ and heated to 350 °C^[^ ^69^ ^]^ by a constant voltage to a Pt heater (R&S HMC803) on the substrate's back side, as monitored with a resistance temperature detector placed on the front of the substrate. The sensor resistance was measured with a multimeter (Keithley 2700) and the response was calculated as

(2) $$S=\frac{R_{\mathrm{air}}}{R_{\mathrm{analyte}}}\;-\;1$$

where *R*_air_ refers to the sensing film resistances in air and *R*_analyte_ to the resistance during analyte exposure. The times needed to reach or recover 90% of the resistance change during or after analyte exposure were defined as response time and recovery time, respectively. Sensing measurements were conducted both with inactive (i.e., with WO_3_ at room temperature; Figure S3a, Supporting Information) and active (i.e., at 240 °C) filter using the gas mixing setup described above and with the additional interferants isoprene (16 ppm), methanol (20 ppm), CO (500 ppm), H_2_ (50 ppm), NO_2_ (10 ppm), and ethylbenzene (10 ppm) as gas standards (all Pan Gas in synthetic air, C*_n_* H*_m_* and NO*_x_* ≤ 100 ppb).

### Handheld Device Integration

As a proof of concept, a miniaturized catalytic packed bed filter and a microsensor utilizing the above nanoparticles were incorporated into a handheld device. Specifically, the miniaturized catalytic filter consisted of 40 mg WO_3_ nanoparticles contained inside a glass tube (i.e., 4 mm inner diameter, 5 cm length, Supelco, Sigma Aldrich) and heated with a resistance wire (0.5 mm diameter, FeCrAl, 6.88 Ω m^−1^) coiled around that tube. The microsensors (1.9  ×  1.7 mm^2^, MSGS 5000i, Microsens SA) were coated with 0.5 mol% Pd/SnO_2_ particles, deposited as described above, and wire‐bonded onto chip carriers.^[^ ^70^ ^]^ Samples were drawn with a pump (Schwarzer Precision, SP 270 EC‐LC 5 VDc), that provided a total flow rate of 100 mL min^−1^, as verified with a bubble flow meter at the pump outlet. All components were connected to a tailor‐made^[^ ^19^ ^]^ PCB hosting also a microcontroller (Raspberry pi Zero W, USA) for automated operation, data acquisition, storage and wireless communication to a smartphone or computer. Power was supplied via a USB port and measurement data were saved on an on‐board secured digital memory card for later evaluation.

### Indoor Air Measurements

Indoor air was collected in Tedlar bags (3L, SKC Inc., USA) and spiked with benzene, toluene and *m*‐xylene from the calibrated gas standards, with a similar protocol applied for methanol elsewhere.^[^ ^71^ ^]^ For analysis, the Tedlar bag was connected first to the PTR‐ToF‐MS and then to the handheld detector for three consecutive exposures of 30 s. Prior to measurements, the detector was three‐point calibrated with synthetic benzene gas mixtures at 40% RH. Benzene concentrations of the spiked samples were quantified by comparison to linear fits of the sensor responses in synthetic air at the same humidity (Figure S14, Supporting Information). A sensor was used to monitor humidity fluctuations (SHT2x, Sensirion AG), while background room air interferants such as ethanol and acetone were monitored by PTR‐ToF‐MS, as elaborated above.

### Statistical Analysis

No preprocessing of the data was done. The mean ± standard deviation (*σ*) were indicated for experiments that were performed under identical conditions with, at least, three replicates. The sample sizes (*n*) for each statistical analysis is indicated. Comparison between the handheld detector and PTR‐ToF‐MS was done by calculating the coefficient of determination *R* ^2^. The device's bias and precision were defined as the average difference and *σ* between the detector and PTR‐ToF‐MS, according to IUPAC.^[^ ^72^ ^]^ The detector calibration curve for the indoor air measurements was obtained by best linear fit. A lognormal fit (Figure S11b, Supporting Information) was applied to calculate the WO_3_ nanoparticle size distribution and determine the geometric average diameter (*d*_g_) and standard deviation (*σ*_g_) using the software OriginPro 2018G (OriginLab Corporation, Massachusetts, USA).

## Conflict of Interest

The authors declare no conflict of interest.

## Author Contributions

I.C.W. and A.T.G. conceived the concept and experiments. I.C.W, P.R., and P.S. performed the experiments. I.C.W., A.T.G. and S.E.P. evaluated the data. A.T.G and S.E.P. provided the funding and were in charge of the project. I.C.W. prepared the first draft. All coauthors revised the manuscript and gave final approval.

## Supporting information

Supporting Information

## Data Availability

The data that support the findings of this study are available from the corresponding author upon reasonable request.
