# Supplementary material for: Handheld Device for Selective Benzene Sensing over Toluene and Xylene
Source: Adv Sci (Weinh). 2021 Nov 27;9(4):2103853. doi: 10.1002/advs.202103853 (PMC8811843; doi:10.1002/advs.202103853)
Supplement: Supplementary file 1 — Supporting Information [file ADVS-9-2103853-s001.pdf]

ADVANCED SCIENCE
Open Access

Supporting Information

for *Adv. Sci.*, DOI: 10.1002/advs.202103853

Handheld device for selective benzene sensing over toluene and xylene

Ines C. Weber, Pascal Rüedi, Petr Šot, Andreas T. Güntner, Sotiris E. Pratsinis

Supporting Information

Handheld device for selective benzene sensing over toluene and xylene

Ines C. Weber, Pascal Rüedi, Petr Šot, Andreas T. Güntner, Sotiris E. Pratsinis

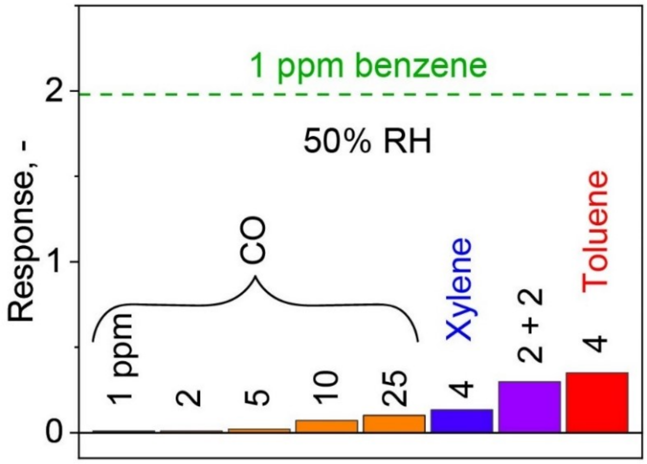

Fig. S1: Benzene selectivity at higher confounder concentrations. Detector responses to 1 – 25 ppm CO (orange), 4 ppm *m*-xylene (blue) and toluene (red), and a mixture of 2 ppm (each) *m*-xylene and toluene (purple) at 50% RH. For comparison, the response to 1 ppm benzene (dashed line) is shown.

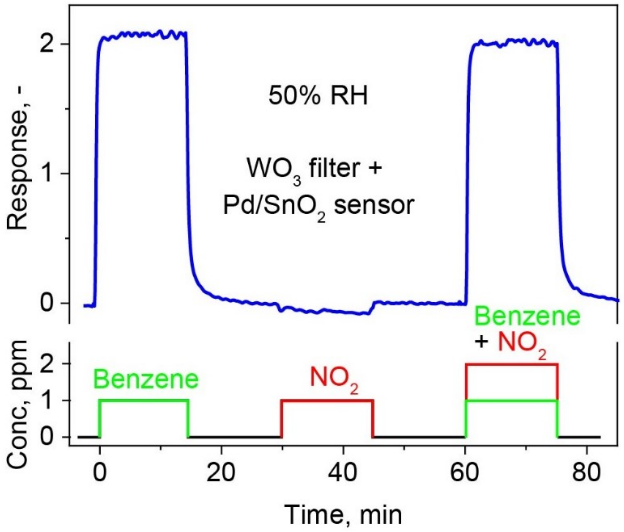

Fig. S2: Benzene selectivity over NO_2. Detector response when exposed subsequently to 1 ppm benzene ($0 \leq t \leq 15$ min), NO_2 ($30 \leq t \leq 45$ min) and their mixture ($60 \leq t \leq 75$ min) at 50% RH.

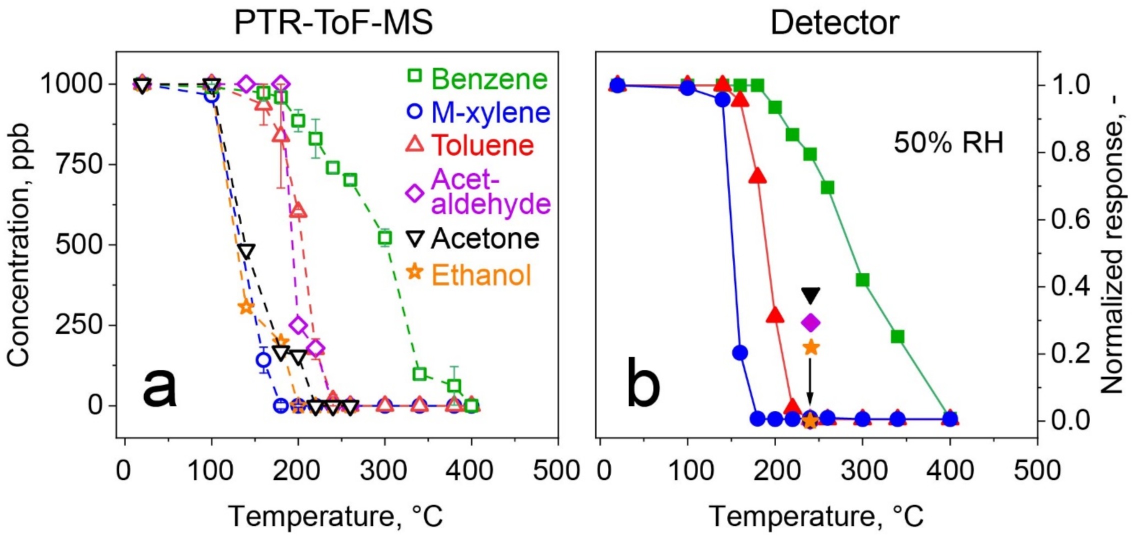

Fig. S3: Conversion on the WO_3 catalyst. Catalytic conversion of 1 ppm benzene (squares), *m*-xylene (circles), toluene (triangles), acetaldehyde (diamonds), acetone (inverse triangles) and ethanol (stars) over WO_3, as quantified by **a)** PTR-ToF-MS and **b)** the sensor as a function of the WO_3 temperature. Error bars for benzene, toluene and *m*-xylene in (a) correspond to n = 3 identically prepared WO_3 particle filters. Note that the sensor response in (b) was normalized to its value at 25 °C for better comparison to PTR-ToF-MS.

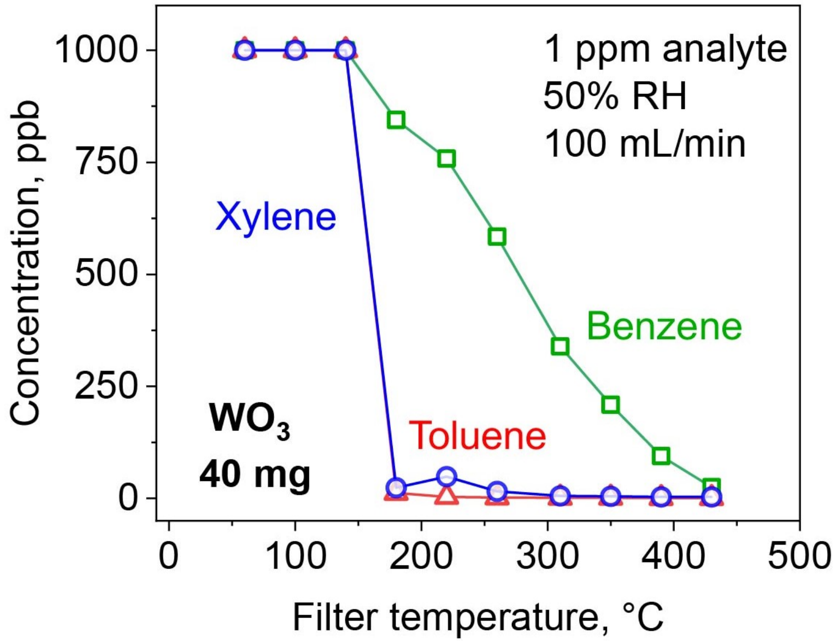

Fig. S4: Catalytic performance of the miniaturized filter. Concentration of 1 ppm *m*-xylene (circles), toluene (triangles) and benzene (squares) after a 40 mg WO_3 filter at 50% RH as a function of its temperature, as measured by PTR-ToF-MS.

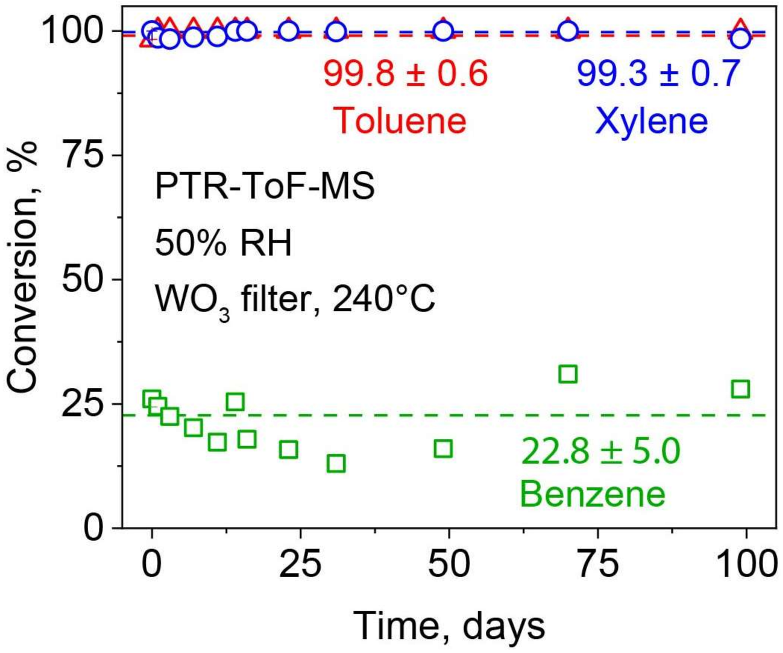

Fig. S5: Catalyst stability. Conversion of a mixture of 1 ppm benzene (squares), toluene (triangles) and *m*-xylene (circles) at 50% RH over a WO_3 filter after storage at ambient condition for 100 days, as measured by PTR-ToF-MS.

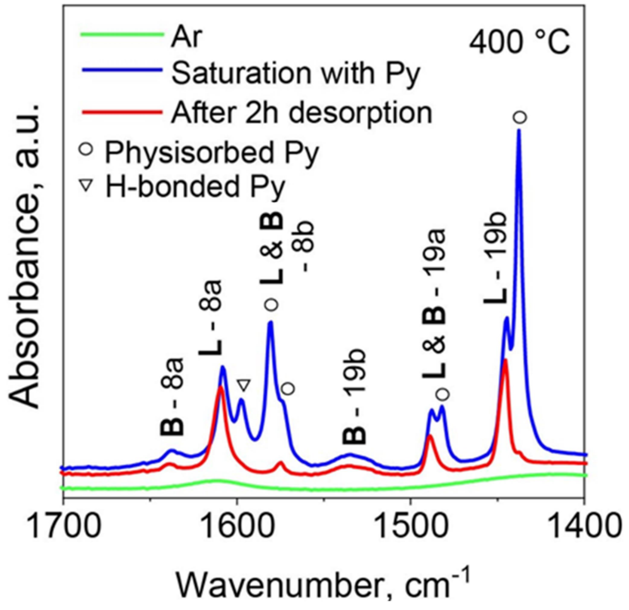

Fig. S6: IR measurements of WO_3 nanoparticles after pyridine adsorption. IR profiles when pretreated at 400 °C under argon (green), saturated with pyridine (blue) and after desorption for 2 h (red). H-bonded pyridine (triangles), physisorbed pyridine (circles) and characteristic vibration modes of Brønsted (**B**) and Lewis (**L**) acid sites are indicated.

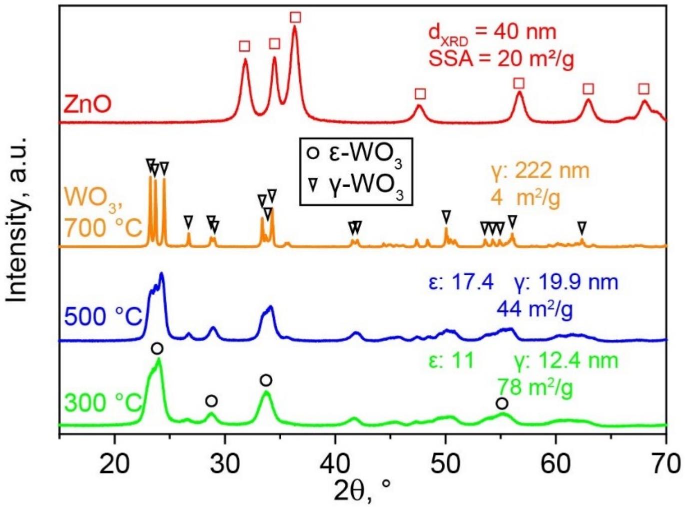

Fig. S7: XRD analysis of WO_3 at different annealing temperatures and ZnO. XRD patterns and reference peaks of the flame-made WO_3 annealed at 300 °C (green), 500 °C (blue), 700 °C (orange) and ZnO (red). Peak locations for monoclinic γ- (triangles), ε-WO_3 (circles) and wurtzite ZnO (squares) are indicated. Crystal sizes (d_{XRD}) as well as N_2 adsorption-measured specific surface areas (SSA) are indicated.

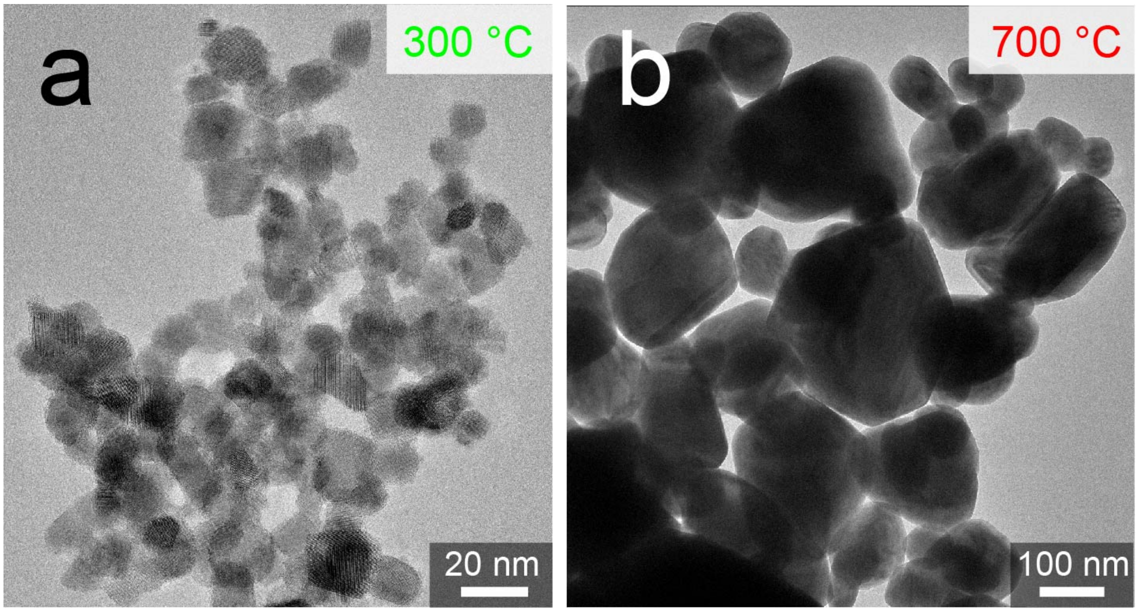

Fig. S8: Morphology characterization of WO_3 at different annealing temperatures. TEM images of WO_3 annealed at **a)** 300 °C and **b)** 700 °C.

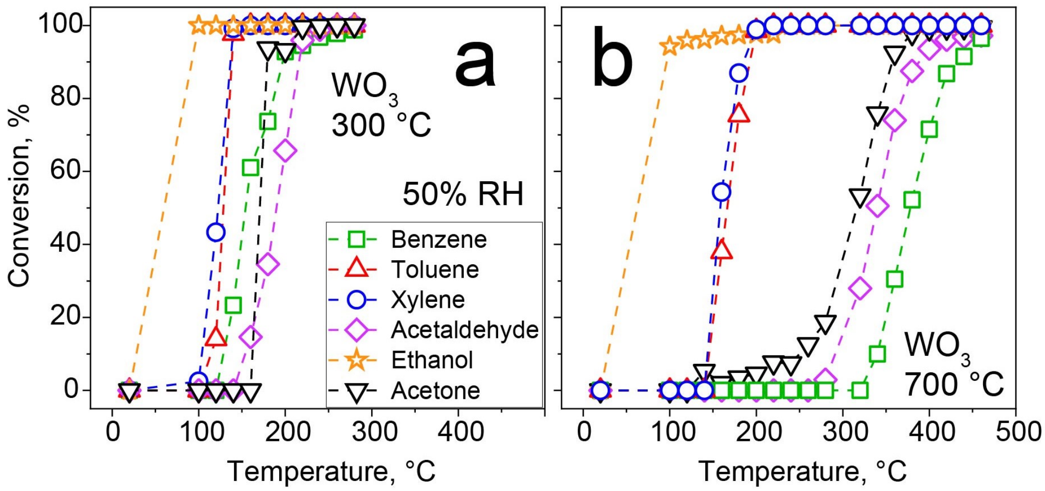

Fig. S9: Catalytic performance of differently annealed WO_3. Catalytic conversion of 1 ppm benzene (squares), *m*-xylene (circles), toluene (triangles), acetaldehyde (diamonds), acetone (inverted triangles) and ethanol (stars) over WO_3 annealed at **a)** 300 °C and **b)** 700 °C at 50% RH, as measured by PTR-ToF-MS.

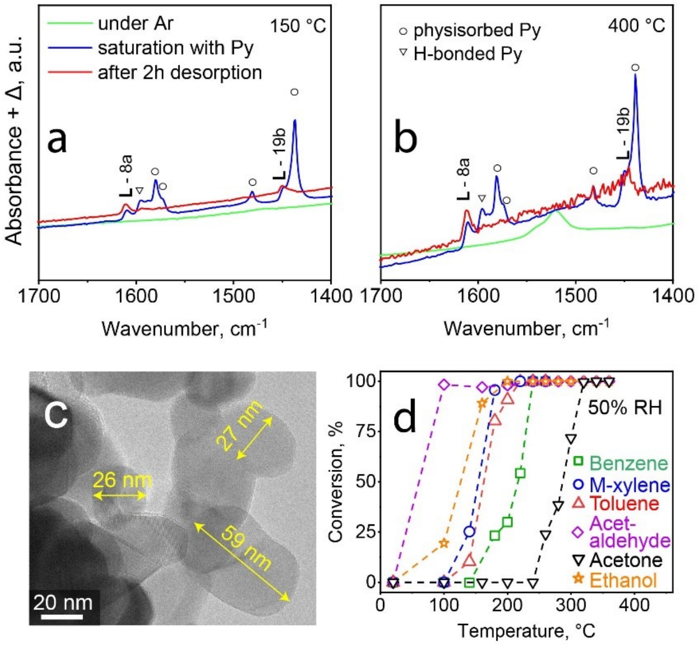

Fig. S10: ZnO material and catalytic characterization. IR profiles when pretreated at 150 °C **a**) and 400 °C **b**) under argon (green), saturated with pyridine (blue) and after desorption for 2 h (red). H-bonded pyridine (triangles), physisorbed pyridine (circles) and characteristic vibration modes of Lewis (**L**) acid sites are indicated. **c)** HRTEM bright field image of the flame-made ZnO particles. Diameters of some particles are indicated. **d)** Catalytic conversion of 1 ppm benzene (squares), *m*-xylene (circles), toluene (triangles), acetaldehyde (diamonds), acetone (inverted triangles) and ethanol (stars) over ZnO at 50% RH, as measured by PTR-ToF-MS. The ZnO catalyst was tested at a catalyst surface area of 3.6 m^2 that is identical to the WO_3.

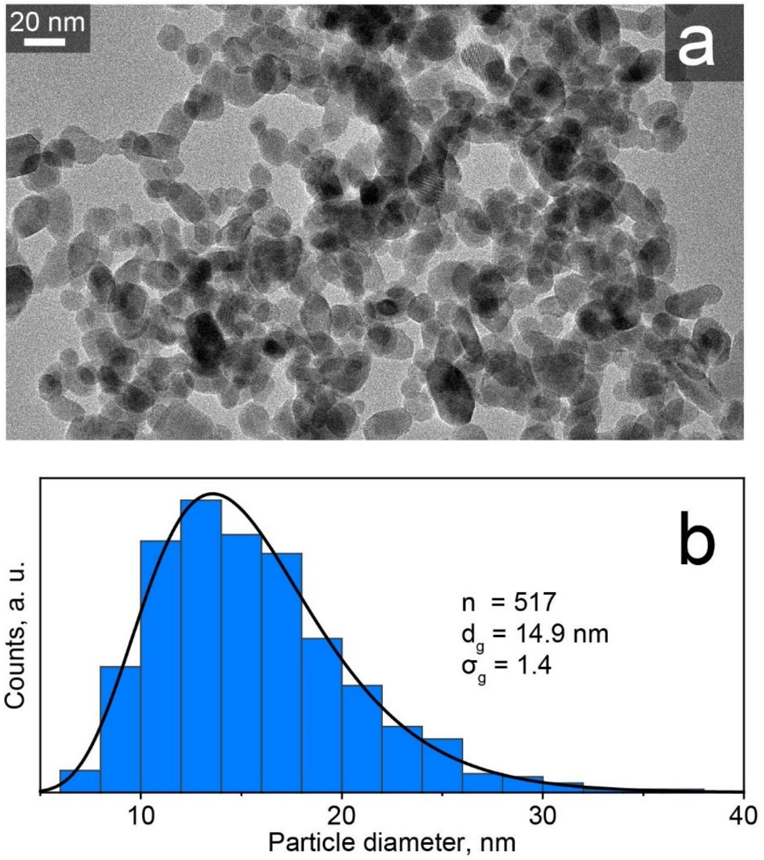

Fig. S11: WO_3 nanoparticle images and size analysis. a) TEM bright field image. **b**) Particle size distribution with a mean geometric diameter (d_g) and respective geometric deviation (σ_g) of n = 517 particles.

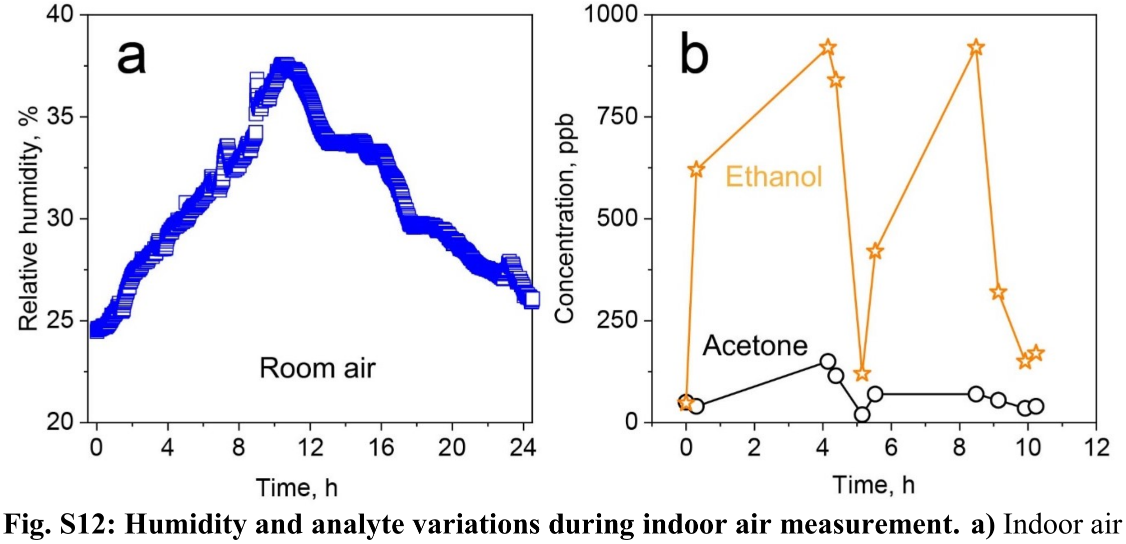

Fig. S12: Humidity and analyte variations during indoor air measurement. a) Indoor air RH as measured with a commercial sensor (Sensirion SHT2x) over 24 h. **b)** Corresponding ethanol (stars) and acetone (circles) concentrations determined by PTR-ToF-MS.

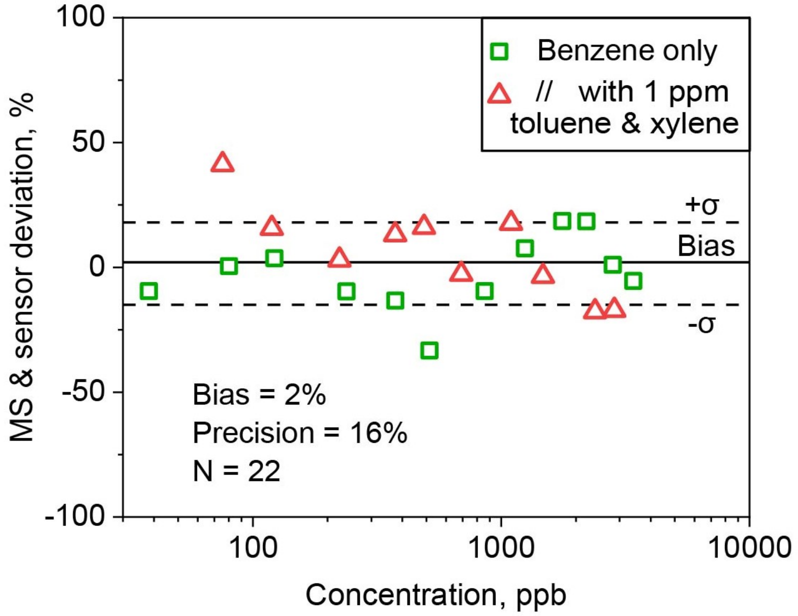

Fig. S13: Detector bias and precision. Relative deviation between the detector and PTR-ToF-MS as a function of the concentration for room air spiked with benzene only (squares) and with additional 1 ppm toluene & *m*-xylene (triangles) for n = 22 samples. Bias and precision (σ) are indicated.

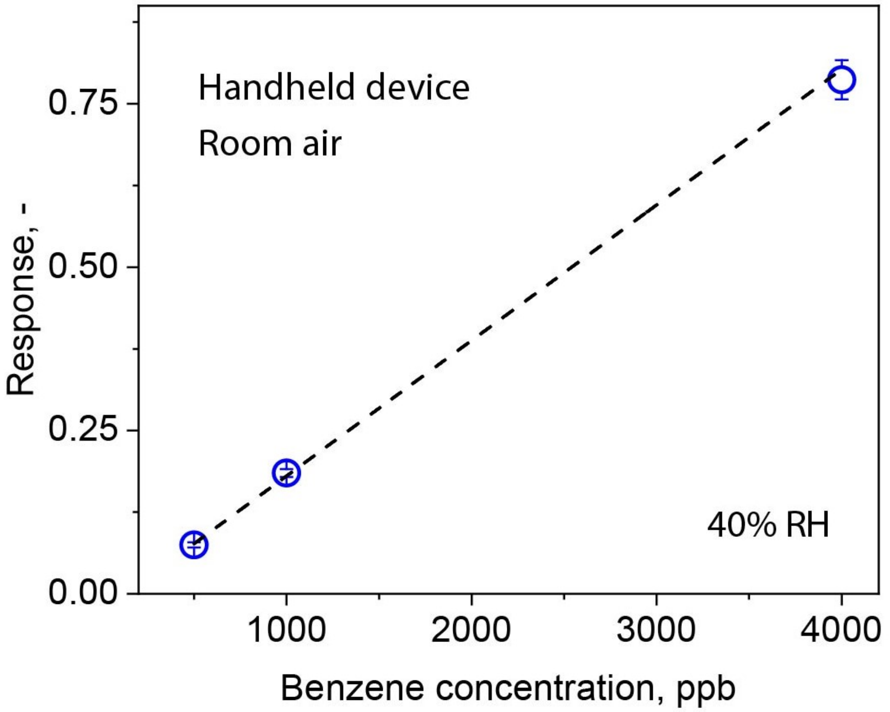

Fig. S14: Hand-held detector calibration. Detector response when exposed to calibrated gas standards at 40% RH. Error bars correspond to n = 3 subsequent measurements.
